# 2-[(*E*)-(5-*tert*-Butyl-2-hydroxy­phen­yl)­diazen­yl]benzoic acid

**DOI:** 10.1107/S160053680803290X

**Published:** 2008-10-18

**Authors:** Tushar S. Basu Baul, Anup Paul, Hadi D. Arman, Edward R. T. Tiekink

**Affiliations:** a Department of Chemistry, North-Eastern Hill University, NEHU Permanent Campus, Umshing, Shillong 793 022, India; b Department of Chemistry, The University of Texas at San Antonio, One UTSA Circle, San Antonio, Texas 78249-0698, USA

## Abstract

The title compound, C_17_H_18_N_2_O_3_, is approximately planar, owing in part to an intra­molecular bifurcated O—H⋯(N,O) hydrogen bond; the dihedral angle between the two aromatic rings is 23.86 (9)°. In the crystal struture, centrosymmetrically related mol­ecules associate into dimers *via* the eight-membered carboxyl­ate {⋯H—O—C=O}_2_ synthon.

## Related literature

For a related structure, see: Basu Baul *et al.* (2007 ▶). For background, see: Willem *et al.* (1998 ▶). For reviews of organotin carboxyl­ates, see: Tiekink (1991 ▶).
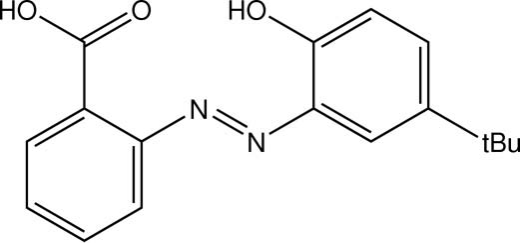


## Experimental

### 

#### Crystal data

C_17_H_18_N_2_O_3_
*M* *_r_* = 298.33
Monoclinic, 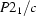

*a* = 5.9052 (19) Å
*b* = 10.872 (4) Å
*c* = 23.126 (8) Å
β = 94.432 (4)°
*V* = 1480.3 (9) Å^3^
*Z* = 4
Mo *K*α radiation
μ = 0.09 mm^−1^
*T* = 98 (2) K
0.35 × 0.16 × 0.08 mm

#### Data collection

Rigaku Saturn724 diffractometer
Absorption correction: none
8445 measured reflections
3068 independent reflections
2610 reflections with *I* > 2σ(*I*)
*R*_int_ = 0.065

#### Refinement

*R*[*F* ^2^ > 2σ(*F* ^2^)] = 0.059
*wR*(*F* ^2^) = 0.144
*S* = 1.12
3068 reflections
205 parameters
2 restraints
H-atom parameters constrained
Δρ_max_ = 0.24 e Å^−3^
Δρ_min_ = −0.31 e Å^−3^

### 

Data collection: *CrystalClear* (Rigaku Americas Corporation, 2005 ▶); cell refinement: *CrystalClear*; data reduction: *CrystalClear*; program(s) used to solve structure: *SHELXS97* (Sheldrick, 2008 ▶); program(s) used to refine structure: *SHELXL97* (Sheldrick, 2008 ▶); molecular graphics: *ORTEPII* (Johnson, 1976 ▶); software used to prepare material for publication: *SHELXL97*.

## Supplementary Material

Crystal structure: contains datablocks global, I. DOI: 10.1107/S160053680803290X/ng2500sup1.cif

Structure factors: contains datablocks I. DOI: 10.1107/S160053680803290X/ng2500Isup2.hkl

Additional supplementary materials:  crystallographic information; 3D view; checkCIF report

## Figures and Tables

**Table 1:** Hydrogen-bond geometry (Å, °)

*D*—H⋯*A*	*D*—H	H⋯*A*	*D*⋯*A*	*D*—H⋯*A*
O3—H3O⋯N1	0.84	1.86	2.587 (2)	144
O3—H3O⋯O1	0.84	2.26	2.894 (2)	132
O2—H2O⋯O1^i^	0.84	1.86	2.687 (2)	170

Symmetry code: (i) 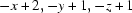.

**Fig. 1.**
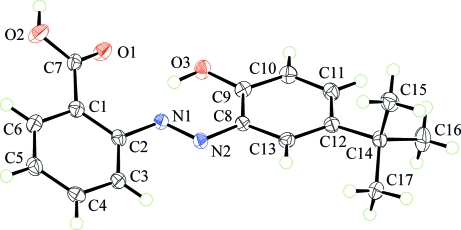
Molecular structure of (I) showing atom-labelling scheme and displacement ellipsoids at the 50% probability level.

## supplementary crystallographic information

### Comment

2-Aminobenzoic acid reacts with 4-*tert*-butyl-phenol to form the title compound (I), Fig. 1, which was prepared during an on-going study of the coordination chemistry of such molecules (Basu Baul *et al.*, 2007) with organotin species (Tiekink, 1991; Willem *et al.*, 1998). The molecule is approximately planar as seen in the value of the dihedral angle formed between the two aromatic residues of 23.86 (9)°. Small twists in the molecule are indicated by the N2–N1–C2–C3 and N1–N2–C8–C9 torsion angles of -18.1 (3) and -2.6 (3)°, respectively. The conformation is stabilized by intramolecular O—H···O and O—H···N hydrogen bonding interactions, Table 1. In the crystal packing, centrosymmetric molecules associate *via* the eight-membered carboxylate {···H—O—C=O}_2_ synthon.

### Experimental

2-Aminobenzoic acid (2.0 g, 14.58 mmol) was mixed with HCl (5 ml) and water (20 ml) and digested in a water bath for 1 h. The hydrochloride was cooled to 5 ° C and diazotized with ice-cold aqueous NaNO_2_ solution (1.1 g, 15.95 mmol, 10 ml). The cold diazonium salt solution was added slowly to 4-*tert*-butyl-phenol (2.19 g, 14.58 mmol), previously dissolved in a NaOH solution (4.0 g) in water (100 ml) under vigorous stirring. A deep-red colour developed almost immediately and stirring was continued for 1 h. The reaction mixture was kept overnight in a refrigerator followed by 2 h at room temperature and then acidified with acetic acid. The brown coloured precipitate was filtered, washed several times with water to remove soluble starting materials, and then dried in air. The dried mass was suspended in water, dissolved in dilute NaOH solution and filtered. The filtrate was collected, precipitated with dilute acetic acid and filtered. The orange-red precipitate was then washed thoroughly with water until the washings were neutral and dried in air. The dried precipitate was boiled in hot hexane, filtered and dried *in vacuo*. Several recrystallization of the precipitate from methanol yielded red plates of (I) (2.50 g, 40.3%), m.pt. 453–455 K. Elemental analysis, found: C 68.54, H 6.01, N 9.49%; C_17_H_18_N_2_O_3_ requires C 68.44, H 6.08, N 9.39%. IR (KBr, cm-1): 1699 ν(OCO)*_asym_*. ^1^H NMR (CDCl_3_, 400.13 MHz, see Fig. 1 for numbering scheme): δ H: 10.7 [br, 2H, OH & CO_2_H], 8.15 [d, 8 Hz, 1H, H6], 7.92 [d, 8 Hz, 1H, H3], 7.78 [d, 2.5 Hz, 1H, H13], 7.58 [t, 8 Hz, 1H, H4], 7.42 [t, 8 Hz, 1H, H5], 7.34 [dd, 2.5, 8 Hz, 1H, H11], 6.96 [d, 8 Hz, 1H, H10], 1.19 [s, 9H, CH_3_] p.p.m. ^13^C NMR (CDCl_3_, 100.62 MHz): δ C: 170.6 [CO_2_], 152.2 [C9], 149.8 [C2], 142.8 [C8], 137.9 [C11], 133.3 [C13], 132.7 [C4], 132.5 [C6], 130.3 [C5], 129.6 [C12'], 125.9 [C1], 119.0 [C10], 116.6 [C3], 34.4 [C14], 31.6 [CH_3_] p.p.m.

### Refinement

All C-bound H atoms were included in the riding-model approximation, with C—H = 0.95 to 0.98 Å, and with *U*_iso_(H) = 1.2–1.5*U*_eq_(C). The hydroxyl-H atom were located from a difference map and included so that O—H = 0.84 Å and *U*_iso_(H) = 1.5*U*_eq_(O).

### Crystal data

**Table:**

C_17_H_18_N_2_O_3_	*F*(000) = 632
*M**_r_* = 298.33	*D*_x_ = 1.339 Mg m^−^^3^
Monoclinic, *P*2_1_/*c*	Mo *K*α radiation, λ = 0.71073 Å
Hall symbol: -P 2ybc	Cell parameters from 7951 reflections
*a* = 5.9052 (19) Å	θ = 2.1–40.6°
*b* = 10.872 (4) Å	µ = 0.09 mm^−^^1^
*c* = 23.126 (8) Å	*T* = 98 K
β = 94.432 (4)°	Prism, red
*V* = 1480.3 (9) Å^3^	0.35 × 0.16 × 0.08 mm
*Z* = 4	

### Data collection

**Table:**

Rigaku Saturn724 (2x2 bin mode) diffractometer	2610 reflections with *I* > 2σ(*I*)
Radiation source: sealed tube	*R*_int_ = 0.065
graphite	θ_max_ = 26.5°, θ_min_ = 2.6°
Detector resolution: 28.5714 pixels mm^-1^	*h* = −7→7
dtprofit.ref scans	*k* = −13→10
8445 measured reflections	*l* = −28→27
3068 independent reflections	

### Refinement

**Table:**

Refinement on *F*^2^	Primary atom site location: structure-invariant direct methods
Least-squares matrix: full	Secondary atom site location: difference Fourier map
*R*[*F*^2^ > 2σ(*F*^2^)] = 0.059	Hydrogen site location: inferred from neighbouring sites
*wR*(*F*^2^) = 0.144	H-atom parameters constrained
*S* = 1.12	*w* = 1/[σ^2^(*F*_o_^2^) + (0.0534*P*)^2^ + 0.6036*P*] where *P* = (*F*_o_^2^ + 2*F*_c_^2^)/3
3068 reflections	(Δ/σ)_max_ < 0.001
205 parameters	Δρ_max_ = 0.24 e Å^−^^3^
2 restraints	Δρ_min_ = −0.31 e Å^−^^3^

### Special details

**Table:**

Geometry. All e.s.d.'s (except the e.s.d. in the dihedral angle between two l.s. planes) are estimated using the full covariance matrix. The cell e.s.d.'s are taken into account individually in the estimation of e.s.d.'s in distances, angles and torsion angles; correlations between e.s.d.'s in cell parameters are only used when they are defined by crystal symmetry. An approximate (isotropic) treatment of cell e.s.d.'s is used for estimating e.s.d.'s involving l.s. planes.
Refinement. Refinement of *F*^2^ against ALL reflections. The weighted *R*-factor *wR* and goodness of fit *S* are based on *F*^2^, conventional *R*-factors *R* are based on *F*, with *F* set to zero for negative *F*^2^. The threshold expression of *F*^2^ > σ(*F*^2^) is used only for calculating *R*-factors(gt) *etc*. and is not relevant to the choice of reflections for refinement. *R*-factors based on *F*^2^ are statistically about twice as large as those based on *F*, and *R*- factors based on ALL data will be even larger.

### Fractional atomic coordinates and isotropic or equivalent isotropic displacement parameters (Å^2^)

**Table:**

	*x*	*y*	*z*	*U*_iso_*/*U*_eq_	
O1	0.8680 (2)	0.51703 (14)	0.43659 (6)	0.0263 (3)	
O2	0.7565 (3)	0.39227 (16)	0.50557 (6)	0.0321 (4)	
H2O	0.8640	0.4274	0.5250	0.048*	
O3	1.0671 (2)	0.59362 (14)	0.33194 (6)	0.0246 (3)	
H3O	0.9599	0.5542	0.3451	0.037*	
N1	0.7361 (2)	0.43713 (16)	0.32732 (6)	0.0190 (3)	
N2	0.7593 (2)	0.40523 (16)	0.27524 (6)	0.0191 (4)	
C1	0.5577 (3)	0.37647 (19)	0.41419 (8)	0.0201 (4)	
C2	0.5548 (3)	0.37664 (18)	0.35309 (8)	0.0185 (4)	
C3	0.3792 (3)	0.31732 (19)	0.32013 (8)	0.0199 (4)	
H3	0.3774	0.3177	0.2790	0.024*	
C4	0.2083 (3)	0.25820 (19)	0.34670 (8)	0.0218 (4)	
H4	0.0892	0.2186	0.3238	0.026*	
C5	0.2102 (3)	0.2566 (2)	0.40711 (8)	0.0243 (4)	
H5	0.0928	0.2157	0.4254	0.029*	
C6	0.3842 (3)	0.3148 (2)	0.44028 (8)	0.0237 (4)	
H6	0.3857	0.3128	0.4814	0.028*	
C7	0.7425 (3)	0.43611 (19)	0.45205 (7)	0.0204 (4)	
C8	0.9426 (3)	0.46168 (18)	0.25033 (7)	0.0179 (4)	
C9	1.0885 (3)	0.55091 (18)	0.27794 (7)	0.0188 (4)	
C10	1.2692 (3)	0.59442 (19)	0.24792 (8)	0.0226 (4)	
H10	1.3672	0.6559	0.2650	0.027*	
C11	1.3068 (3)	0.54898 (19)	0.19368 (8)	0.0218 (4)	
H11	1.4315	0.5800	0.1745	0.026*	
C12	1.1679 (3)	0.45878 (19)	0.16583 (8)	0.0195 (4)	
C13	0.9861 (3)	0.41778 (19)	0.19516 (7)	0.0201 (4)	
H13	0.8872	0.3577	0.1772	0.024*	
C14	1.2258 (3)	0.4010 (2)	0.10821 (8)	0.0221 (4)	
C15	1.3827 (3)	0.2902 (2)	0.12276 (9)	0.0285 (5)	
H15A	1.4224	0.2510	0.0867	0.043*	
H15B	1.3040	0.2306	0.1459	0.043*	
H15C	1.5213	0.3183	0.1449	0.043*	
C16	1.3498 (4)	0.4929 (2)	0.07107 (9)	0.0350 (5)	
H16A	1.3845	0.4533	0.0347	0.052*	
H16B	1.4913	0.5194	0.0924	0.052*	
H16C	1.2524	0.5646	0.0624	0.052*	
C17	1.0117 (3)	0.3565 (2)	0.07259 (8)	0.0300 (5)	
H17A	1.0544	0.3198	0.0363	0.045*	
H17B	0.9099	0.4263	0.0639	0.045*	
H17C	0.9341	0.2948	0.0948	0.045*	

### Atomic displacement parameters (Å^2^)

**Table:**

	*U*^11^	*U*^22^	*U*^33^	*U*^12^	*U*^13^	*U*^23^
O1	0.0322 (7)	0.0266 (8)	0.0198 (6)	−0.0063 (6)	−0.0008 (5)	0.0028 (6)
O2	0.0389 (8)	0.0371 (10)	0.0193 (7)	−0.0135 (7)	−0.0048 (6)	0.0059 (6)
O3	0.0284 (7)	0.0242 (8)	0.0218 (6)	−0.0050 (6)	0.0052 (5)	−0.0054 (6)
N1	0.0187 (7)	0.0213 (9)	0.0171 (7)	0.0019 (6)	0.0024 (5)	0.0007 (6)
N2	0.0185 (7)	0.0208 (9)	0.0181 (7)	0.0017 (6)	0.0029 (6)	0.0004 (6)
C1	0.0215 (9)	0.0184 (10)	0.0208 (9)	0.0024 (7)	0.0033 (7)	−0.0002 (7)
C2	0.0187 (8)	0.0165 (9)	0.0207 (8)	0.0032 (7)	0.0045 (6)	0.0008 (7)
C3	0.0208 (8)	0.0192 (10)	0.0200 (8)	0.0044 (7)	0.0031 (7)	−0.0011 (7)
C4	0.0182 (8)	0.0196 (10)	0.0274 (9)	0.0014 (7)	0.0009 (7)	−0.0014 (8)
C5	0.0222 (9)	0.0263 (11)	0.0252 (9)	−0.0010 (8)	0.0067 (7)	0.0047 (8)
C6	0.0283 (9)	0.0244 (11)	0.0185 (8)	0.0016 (8)	0.0034 (7)	0.0005 (8)
C7	0.0240 (9)	0.0210 (10)	0.0166 (8)	0.0024 (8)	0.0041 (7)	0.0001 (7)
C8	0.0173 (8)	0.0188 (10)	0.0177 (8)	0.0006 (7)	0.0021 (6)	0.0011 (7)
C9	0.0217 (8)	0.0152 (9)	0.0194 (8)	0.0016 (7)	0.0010 (6)	−0.0010 (7)
C10	0.0240 (9)	0.0184 (10)	0.0254 (9)	−0.0033 (7)	0.0018 (7)	0.0003 (8)
C11	0.0203 (8)	0.0201 (10)	0.0256 (9)	−0.0014 (7)	0.0050 (7)	0.0043 (8)
C12	0.0200 (8)	0.0187 (10)	0.0199 (8)	0.0019 (7)	0.0025 (6)	0.0021 (7)
C13	0.0198 (8)	0.0215 (10)	0.0189 (8)	−0.0016 (7)	0.0006 (7)	−0.0011 (7)
C14	0.0212 (9)	0.0260 (11)	0.0197 (9)	−0.0016 (8)	0.0060 (7)	0.0003 (8)
C15	0.0262 (9)	0.0303 (12)	0.0292 (10)	0.0024 (8)	0.0040 (8)	−0.0069 (9)
C16	0.0397 (12)	0.0378 (14)	0.0295 (10)	−0.0080 (10)	0.0157 (9)	0.0012 (10)
C17	0.0250 (9)	0.0468 (15)	0.0185 (9)	0.0007 (9)	0.0039 (7)	−0.0049 (9)

### Geometric parameters (Å, °)

**Table:**

O1—C7	1.221 (2)	C9—C10	1.400 (3)
O2—C7	1.323 (2)	C10—C11	1.382 (3)
O2—H2O	0.84	C10—H10	0.9500
O3—C9	1.348 (2)	C11—C12	1.403 (3)
O3—H3O	0.84	C11—H11	0.9500
N1—N2	1.271 (2)	C12—C13	1.387 (2)
N1—C2	1.426 (2)	C12—C14	1.536 (3)
N2—C8	1.406 (2)	C13—H13	0.9500
C1—C6	1.400 (3)	C14—C17	1.533 (3)
C1—C2	1.412 (2)	C14—C16	1.539 (3)
C1—C7	1.494 (3)	C14—C15	1.541 (3)
C2—C3	1.396 (3)	C15—H15A	0.9800
C3—C4	1.380 (3)	C15—H15B	0.9800
C3—H3	0.9500	C15—H15C	0.9800
C4—C5	1.396 (3)	C16—H16A	0.9800
C4—H4	0.9500	C16—H16B	0.9800
C5—C6	1.387 (3)	C16—H16C	0.9800
C5—H5	0.9500	C17—H17A	0.9800
C6—H6	0.9500	C17—H17B	0.9800
C8—C13	1.404 (2)	C17—H17C	0.9800
C8—C9	1.417 (3)		
			
C7—O2—H2O	109.0	C10—C11—C12	122.50 (17)
C9—O3—H3O	106.6	C10—C11—H11	118.7
N2—N1—C2	114.19 (16)	C12—C11—H11	118.7
N1—N2—C8	114.34 (15)	C13—C12—C11	116.58 (17)
C6—C1—C2	118.67 (17)	C13—C12—C14	121.71 (17)
C6—C1—C7	118.77 (16)	C11—C12—C14	121.52 (16)
C2—C1—C7	122.54 (16)	C12—C13—C8	122.54 (17)
C3—C2—C1	119.78 (17)	C12—C13—H13	118.7
C3—C2—N1	122.33 (16)	C8—C13—H13	118.7
C1—C2—N1	117.88 (16)	C17—C14—C12	111.46 (15)
C4—C3—C2	120.65 (17)	C17—C14—C16	108.24 (17)
C4—C3—H3	119.7	C12—C14—C16	111.47 (17)
C2—C3—H3	119.7	C17—C14—C15	109.10 (18)
C3—C4—C5	120.12 (17)	C12—C14—C15	107.52 (15)
C3—C4—H4	119.9	C16—C14—C15	109.01 (17)
C5—C4—H4	119.9	C14—C15—H15A	109.5
C6—C5—C4	119.72 (18)	C14—C15—H15B	109.5
C6—C5—H5	120.1	H15A—C15—H15B	109.5
C4—C5—H5	120.1	C14—C15—H15C	109.5
C5—C6—C1	121.06 (17)	H15A—C15—H15C	109.5
C5—C6—H6	119.5	H15B—C15—H15C	109.5
C1—C6—H6	119.5	C14—C16—H16A	109.5
O1—C7—O2	122.55 (17)	C14—C16—H16B	109.5
O1—C7—C1	124.99 (16)	H16A—C16—H16B	109.5
O2—C7—C1	112.46 (17)	C14—C16—H16C	109.5
C13—C8—N2	115.13 (16)	H16A—C16—H16C	109.5
C13—C8—C9	119.64 (16)	H16B—C16—H16C	109.5
N2—C8—C9	125.11 (16)	C14—C17—H17A	109.5
O3—C9—C10	118.24 (17)	C14—C17—H17B	109.5
O3—C9—C8	123.79 (16)	H17A—C17—H17B	109.5
C10—C9—C8	117.94 (17)	C14—C17—H17C	109.5
C11—C10—C9	120.77 (18)	H17A—C17—H17C	109.5
C11—C10—H10	119.6	H17B—C17—H17C	109.5
C9—C10—H10	119.6		
			
C2—N1—N2—C8	−178.01 (15)	C13—C8—C9—O3	−176.45 (17)
C6—C1—C2—C3	0.8 (3)	N2—C8—C9—O3	−0.7 (3)
C7—C1—C2—C3	178.85 (18)	C13—C8—C9—C10	1.6 (3)
C6—C1—C2—N1	−178.37 (17)	N2—C8—C9—C10	177.41 (17)
C7—C1—C2—N1	−0.4 (3)	O3—C9—C10—C11	176.50 (17)
N2—N1—C2—C3	−18.1 (3)	C8—C9—C10—C11	−1.7 (3)
N2—N1—C2—C1	161.07 (17)	C9—C10—C11—C12	0.4 (3)
C1—C2—C3—C4	−0.1 (3)	C10—C11—C12—C13	1.0 (3)
N1—C2—C3—C4	179.05 (17)	C10—C11—C12—C14	−174.16 (18)
C2—C3—C4—C5	−0.4 (3)	C11—C12—C13—C8	−1.0 (3)
C3—C4—C5—C6	0.1 (3)	C14—C12—C13—C8	174.10 (17)
C4—C5—C6—C1	0.6 (3)	N2—C8—C13—C12	−176.47 (17)
C2—C1—C6—C5	−1.1 (3)	C9—C8—C13—C12	−0.3 (3)
C7—C1—C6—C5	−179.17 (18)	C13—C12—C14—C17	32.4 (3)
C6—C1—C7—O1	−160.29 (19)	C11—C12—C14—C17	−152.69 (19)
C2—C1—C7—O1	21.7 (3)	C13—C12—C14—C16	153.50 (18)
C6—C1—C7—O2	19.2 (3)	C11—C12—C14—C16	−31.6 (3)
C2—C1—C7—O2	−158.82 (18)	C13—C12—C14—C15	−87.1 (2)
N1—N2—C8—C13	173.37 (16)	C11—C12—C14—C15	87.8 (2)
N1—N2—C8—C9	−2.6 (3)		

### Hydrogen-bond geometry (Å, °)

**Table:**

*D*—H···*A*	*D*—H	H···*A*	*D*···*A*	*D*—H···*A*
O3—H3O···N1	0.84	1.86	2.587 (2)	144
O3—H3O···O1	0.84	2.26	2.894 (2)	132
O2—H2O···O1^i^	0.84	1.86	2.687 (2)	170

Symmetry codes:  (i) −*x*+2, −*y*+1, −*z*+1.
